# EGF Enhances Oligodendrogenesis from Glial Progenitor Cells

**DOI:** 10.3389/fnmol.2017.00106

**Published:** 2017-04-11

**Authors:** Junlin Yang, Xuejun Cheng, Jiajun Qi, Binghua Xie, Xiaofeng Zhao, Kang Zheng, Zunyi Zhang, Mengsheng Qiu

**Affiliations:** ^1^The Institute of Developmental and Regenerative Biology, Zhejiang Key Laboratory of Organ Development and Regeneration, College of Life and Environment Sciences, Hangzhou Normal UniversityHangzhou, China; ^2^Department of Anatomical Sciences and Neurobiology, University of LouisvilleLouisville, KY, USA

**Keywords:** GRP cell, OPC, oligodendrocyte lineage, self-renewal, synergistic effect

## Abstract

Emerging evidence indicates that epidermal growth factor (EGF) signaling plays a positive role in myelin development and repair, but little is known about its biological effects on the early generation and differentiation of oligodendrocyte (OL) lineage cells. In this study, we investigated the role of EGF in early OL development with isolated glial restricted precursor (GRP) cells. It was found that EGF collaborated with Platelet Derived Growth Factor-AA (PDGFaa) to promote the survival and self-renewal of GRP cells, but predisposed GRP cells to develop into O4^−^ early-stage oligodendrocyte precursor cells (OPCs) in the absence of or PDGFaa. In OPCs, EGF synergized with PDGFaa to maintain their O4 negative antigenic phenotype. Upon PDGFaa withdrawal, EGF promoted the terminal differentiation of OPCs by reducing apoptosis and increasing the number of mature OLs. Together, these data revealed that EGF is an important mitogen to enhance oligodendroglial development.

## Introduction

Oligodendrocytes (OLs) elaborate insulating myelin sheaths that wrap around axons to ensure the rapid conduction of nerve impulses and axonal survival (Qiu, 2013; Zhang et al., 2013; Blank and Prinz, 2014; Alizadeh et al., 2015; Rao and Pearse, 2016). Tripotential glial-restricted precursor (GRP) cells were initially found in embryonic spinal tissues (Herrera et al., 2001; Gregori et al., 2002; Wu et al., 2002; Cao et al., 2005; Phillips et al., 2012), as they can generate OLs and type I and type II astrocytes *in vitro* (Rao et al., 1998; Gregori et al., 2002; Yang et al., 2016) and *in vivo* (Herrera et al., 2001; Hill et al., 2004). The germination of GRP cells from neuroepithelial stem cells was viewed as the beginning of OL generation (Rao et al., 1998; Gregori et al., 2002). Tripotential GRP cells then generate bipotential oligodendrocyte precursor cells (OPCs) which are capable of differentiating into either OLs or type II astrocytes (Morath and Mayer-Pröschel, 2001; Gregori et al., 2002). OPCs proliferate and migrate throughout the CNS during late mouse embryonic development, and later differentiate into mature myelinating OLs (Fernandez et al., 2004; Cai et al., 2010; Chen et al., 2015).

Emerging evidence suggests that epidermal growth factor (EGF) signaling plays an important role in oligodendroglial development (Aguirre et al., 2007; Chong et al., 2008; Hu et al., 2010). Loss-of-function of epidermal growth factor receptor (EGFR) reduced oligodendrogenesis *in vivo* (Aguirre et al., 2007); conversely, intraventricular infusion of EGF induced subventricular zone (SVZ) type B cells to migrate and differentiate into OLs (Gonzalez-Perez et al., 2009). More recently, it was shown that intranasal EGF treatment immediately after brain injury promoted oligodendrogenesis and remyelination (Scafidi et al., 2014). Although the importance of EGF signaling in the development of OL lineage has been established, it remains elusive at what stage of oligodendrogenesis EGF starts to function and how it regulates the development of OL lineage progression.

In this study, we used mouse GRP cells as the starting point to systematically investigate the role of EGF signaling in OL lineage development. It was found that cells of OL lineage were responsive to EGF at all developmental stages. In GRP cells, EGF promoted their proliferation and survival by augmenting their responsiveness to Platelet Derived Growth Factor-AA (PDGFaa) for self-renewal. In the absence of PDGFaa, EGF predisposed GRP cells to differentiate into O4^−^ early-stage OPCs. At OPC stage, EGF collaborated with PDGFaa to enhance OPC self-renewal. Upon PDGFaa withdrawal, OPCs underwent terminal differentiation and EGF functioned to reduce apoptosis and increase the number of mature OLs.

## Materials and Methods

### Isolation and Culture of GRP Cells

GRP cells were isolated from E13.5 C57BL/6 mouse spinal cord by A2B5 immunopanning as described elsewhere (Gregori et al., 2002), all experimental procedures were carried out in accordance with institutional guidelines for the care and use of laboratory animals, and the protocol was approved by the Animal Ethics Committee of Hangzhou Normal University, China. A2B5^+^ cells were then grown in glial basal medium (DMEM/F12, 1 × N2, 1 × B27, 1 × P/S, and 0.1% w/v BSA, all from Gibco) supplemented with 10 ng/ml PDGFaa and EGF (Peprotech) on fibronectin/laminin coated 12-well plates at 2000 cells/well for mass culture experiments or on coated 24-well plated at 1000 cells/well for immunofluorescence staining. The immunostaining was performed 3 days after seeding using the standard protocols.

### Clonal Analysis and Sub-Cloning of GRP Cells

Immunopurified A2B5^+^ cells from E13.5 spinal tissues were adjusted to a cell density of 10 cells/ml with glial basal medium supplemented with 10 ng/ml EGF and PDGFaa, then the cell suspension was added into fibronectin/laminin-coated 96-well plates at 100 μl/well, and wells with a single cell were marked for further culture. When primary clones were generated, 10 clones were randomly selected and only one was found to be A2B5 negative. Of the nine A2B5^+^ clones, three were subjected to differentiation potential analysis as indicated in the "Results" Section. The other six A2B5^+^ clones were used for sub-cloning analysis. Each clone was replated on three separate grid dishes at equal clonal density, and cultured in presence of EGF, PDGFaa or both. After 6 days, the numbers of secondary clones were scored.

### Cell Proliferation and Apoptosis Analysis

For cell proliferation and apoptosis analysis, 1 × 10^4^ cells were plated to each fibronectin/laminin-coated 24-well plates. Cell proliferation was analyzed by adding BrdU (Sigma) to a final concentration of 30 ng/ml. Following 24 h of incorporation, cells were fixed in 4% paraformaldehyde at RT for 10 min, and BrdU positive cells were detected by anti-BrdU immunostaining. For apoptosis assays, cells were fixed in 4% paraformaldehyde 3 days after replating and apoptotic cells were detected by TUNEL FITC Apoptosis Detection Kit (Vazyme Biotech). Positive cells were counted from three different areas of each well under fluorescence microscopy. The results were expressed as mean values and standard deviation.

### GRP Cell Development

The function of EGF on the development of GRP cells into OPCs was confirmed by single-cell tracking clonal differentiation analysis as described previously (Gregori et al., 2002). Freshly immunopurified GRP cells from E13.5 spinal tissues were adjusted to a cell density of 10 cells/ml with glial basal medium supplemented, then the cell suspension was added into fibronectin/laminin-coated 48-well plates at 100 μl/well in the presence of EGF and PDGFaa (10 ng/ml) for 24 h before being exposed to the factors as indicated in the "Results" Section wells with a single cell were marked for further culture. Since there is no specific immunological marker to distinguish GRP cells from OPCs, one candidate marker for distinguishing them is the O4 monoclonal antibody, which labels late-stage OPCs and OLs (Gard and Pfeiffer, 1990; Bansal et al., 1992). Therefore, the proportion of O4^+^ cells was used as a standard to estimate the differentiation of GRP cells.

### OPC Culture

O4^−^ early-stage OPCs were induced from GRP cells and plated on the fibronectin/laminin-coated plates and fed every other day with glial basal medium supplemented with EGF and PDGFaa. Because of the presence of contact inhibition, OPCs were plated more sparsely than GRP cells, and passaged more frequently. OPCs were plated at 1500 cells/cm^2^ for mass culture, and 750 cells/cm^2^ for differentiation experiments due to process growth.

### Western Immunoblotting

Western blotting was carried out as previously described (Yang et al., 2009). Briefly, cells were lysed in sample buffer plus a cocktail of protease inhibitors (Roche). For each sample, 20 μg of protein was used for electrophoresis in SDS-PAGE gel. Primary antibodies were used as follows; anti-rabbit PDGFRα (1:1000, Santa Cruz), anti- rabbit EGFR (1:200, Abcam), anti- rabbit Olig2 (1:1000, Millipore), anti-rabbit Nestin (1:5000, Covance) and anti-mouse MBP (1:1000, Abcam). Horseradish peroxidase (HRP)-conjugated secondary antibody (Promaga) was used at 1:2500. Chemiluminescent signals were detected by autoradiography using the ECL System (Amersham, Piscataway, NJ, USA).

### Immunocytochemical Analysis

Immunocytochemical analysis was carried out as previously described (Cheng et al., 2017). Antibodies used include anti-mouse A2B5 IgM, anti-BrdU IgM, O4 IgM (1:1 dilution in DPBS + 10% goat serum), anti-mouse Olig2 (1:1000, Millipore), anti-mouse MBP (1:500, Abcam), anti- rabbit EGFR (1:200, Abcam), anti-mouse GFAP (1:300, Chemicon), anti-rabbit Nestin (1:2000, Covance), and anti-rabbit neurofilament (1:100, Sigma). The Alexa-488 or Alexa-594 conjugated secondary antibodies were obtained from Molecular Probes (Thermo fisher). The nucleic acid dye 4′,6-diamidino-2-phenylindole (DAPI) was obtained from Roche.

### Statistical Analysis

All quantitative data are presented as means ± SD. Statistical significance of the difference was evaluated by Student’s *t*-test. *P*-value < 0.05 was considered statistically significant.

## Results

### EGF Enhances the Survival and Extensive Self-Renewal of GRP Cells in Culture

**Figure 1:**
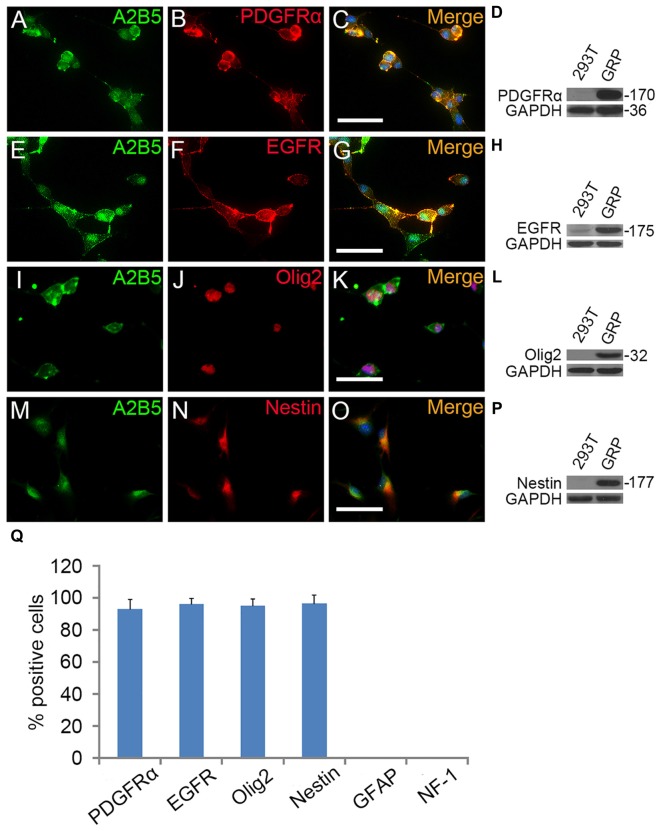
**The antigenic phenotypes of E13.5 spinal cord derived A2B5^+^ cells.** **(A–P)** Immunostaining of A2B5^+^ cells with PDGFRα, epidermal growth factor receptor (EGFR), Olig2 and Nestin antibodies, respectively, and the antigenic phenotypes were confirmed by Western blotting **(D,H,L,P)**. **(Q)** Quantification of PDGFRα^+^, EGFR^+^, Olig2^+^, Nestin^+^, GFAP^+^ and NF-1^+^ cells in A2B5^+^ cell cultures, *n* = 3. Scale bars: 50 μm.

**Figure 2:**
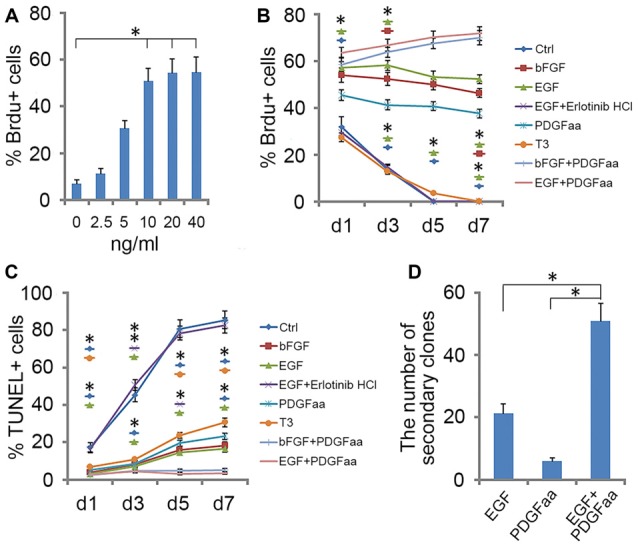
**Effect of EGF on A2B5^+^ cells. (A)** Histogram of the number of BrdU^+^ cells found in A2B5^+^ cell cultures after exposure to different doses of EGF. *Indicates differences between groups 10, 20 and 40 ng/ml EGF vs. the low-dose groups, *P* < 0.05. No differences were found among the groups of 10, 20 and 40 ng/ml. *n* = 3. **(B,C)** Quantification of BrdU^+^ and TUNEL^+^ cells in A2B5^+^ cell cultures after EGF, EGF + Erlotinib-HCl, basic fibroblast growth factor (bFGF), PDGFaa, T3, bFGF + PDGFaa and EGF + PDGFaa treatments for various time lengths, Ctrl refers to the groups without any supplemented factor, *n* = 3. **(D)** Histogram of the number of secondary clones in A2B5^+^ cell cultures at clonal density after exposure to EGF, PDGFaa and EGF + PDGFaa, respectively. *n* = 3. Statistical analyses are presented as mean ± SD. *P < 0.05, **P < 0.01.

**Figure 3:**
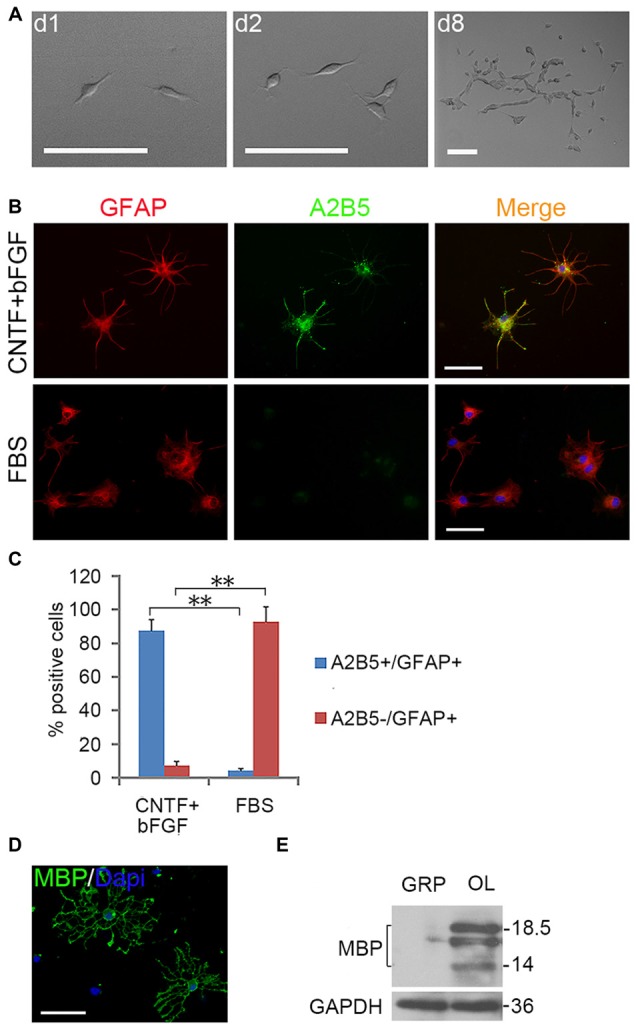
**Expanded clones of A2B5^+^ cell expressed typical differentiation phenotype of tripotential glial restricted precursor (GRP) cells. (A)** An example of an expanded clone in presence of EGF + PDGFaa. **(B)** A2B5^+^ clones were digested and replated into separate wells of 24-well plates and induced to differentiate for 5 days in the presence of bFGF + CNTF or FBS, A2B5^+^/GFAP^+^ and A2B5^−^/GFAP^+^ astrocytes were obtained, respectively. **(C)** Quantification of A2B5^+^/GFAP^+^ and A2B5^−^/GFAP^+^ astrocytes in the differentiation cultures exposed to bFGF + CNTF and FBS, *n* = 3. **(D)** A2B5^+^ cells were cultured in T3 for 5 days and MBP^+^ Oligodendrocytes (OLs) can be detected. **(E)** The culture described in **(D)** was confirmed by western blotting with MBP antibody. Abbreviation: OL, oligodendrocyte. **P < 0.01. Scale bars: **(A)** 100 μm; **(B,D)** 50 μm.

**Figure 4:**
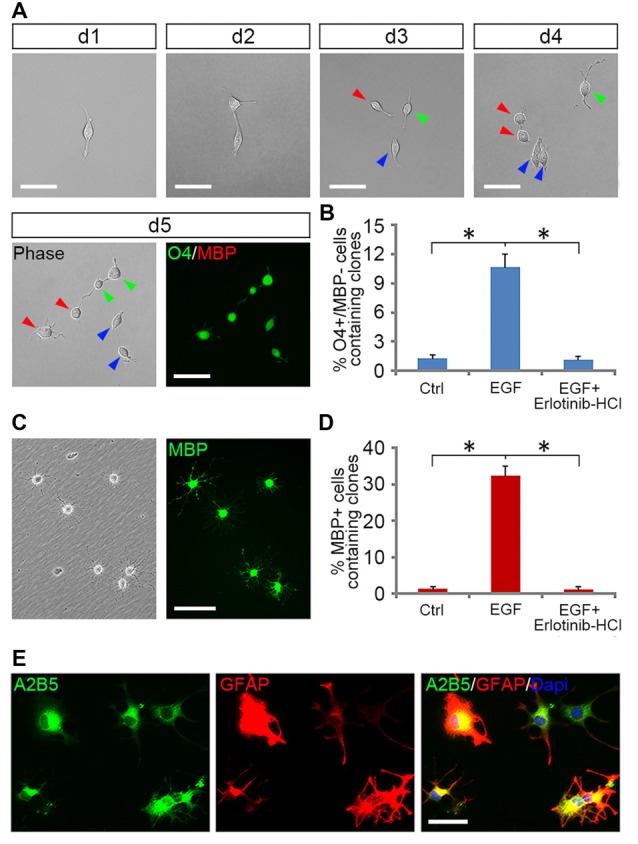
**EGF enhanced the formation of OPCs from GRP clones. (A)** The single cell tracking of a GRP cell generating O4^+^/MBP^−^ daughter cells in EGF for 5 days. With the progression of cell division, a fibroblast-like GRP cell was gradually converted to typical OPCs of bipolar or tripolar morphology, well separated from each other, and expressed O4 antigen but not MBP. Daughter cells of the same progenitor were indicated by arrows of different color. **(B)** Quantification of the clones containing O4^+^/MBP^−^ cells in the GRP cell cultures exposed to various combinations of factors for 5 days, respectively, *n* = 3. **(C)** OPC-like cells from **(B)** differentiated into MBP^+^ cells after 5 days of culture in glial basal medium without supplemented factors. Left: phase image; right: anti-MBP immunostaining. **(D)** Quantification of the clones containing MBP^+^ cells in OL differentiation cultures described in **(C)**, *n* = 3. **(E)** OPCs derived from EGF + PDGFaa + T3 treament differentiated into A2B5^+^/GFAP^+^ astrocytes in presence of FBS. *P < 0.05. Scale bars: **(A,C)** 75 μm; (**E**) 50 μm.

To investigate the role of EGF on OL lineage development, we first immunopurified A2B5^+^ cells from E13.5 spinal cord tissues (Liu et al., 2002). These A2B5^+^ cells expressed typical GRP markers including PDGFRα (Rao et al., 1998), Olig2 (Zhao et al., 2009) and Nestin (Yoo and Wrathall, 2007; Figures 1A–D,I–Q), but not neuronal marker neurofilament (Tang et al., 2007; Rao and Pearse, 2016) and astrocyte marker GFAP (Sun et al., 2008; Sántha et al., 2016). The GRP cells were also immunoreactive for EGFR (Figures 1E–H,Q) suggesting their potential EGF-responsiveness. The effects of EGF on A2B5^+^ cell proliferation were investigated in culture exposed to different concentrations of EGF (0, 2.5, 5, 10, 20 and 40 ng/ml) for 24 h. BrdU labeling revealed a dose-dependent effect on cell division at the concentration range of 0–10 ng/ml (Figure 2A). However, no statistical differences were found in the percentage of BrdU^+^ cells among the 10, 20 and 40 ng/ml EGF groups (Figure 2A), indicating that cell proliferation plateaued at 10 ng/ml. When we grew A2B5^+^ cells for 7 days, EGF was found to promote the cell proliferation and survival as effectively as basic fibroblast growth factor (bFGF), but the percentages of BrdU^+^ cells on d3 and d7 in EGF groups were higher than those of bFGF groups (Figures 2B,C). When GRP cells were treated with EGF and Erlotinib HCl (an antagonist of EGFR) simultaneously, the biological effects of EGF on promoting the division and survival was neutralized (Figures 2B,C), and the antigenic phenotypes were similar to those of the control (Figure 4B). T3 did not significantly enhance the proliferation of A2B5^+^ cells compared to control group (Figure 2B), but it significantly reduced cell apoptosis (Figure 2C), probably by promoting A2B5^+^ cell differentiation into OLs (Figures 3D,E).

EGF also cooperated with PDGFaa to promote a vibrant proliferation of A2B5^+^ cells as bFGF + PDGFaa did (Figures 2B,C). As a result, EGF + PDGFaa stimulated A2B5^+^ cells to divide continuously and form clones (Figures 2B, 3A). Three randomly chosen primary clones (EGF + PDGFaa treatment) were digested into single cells with trypsin and replated for antigen phenotyping and differentiation potential analysis. All clones from the freshly immunopurified A2B5^+^ cells expressed the same antigens as described above. Cells grown in the presence of cholinergic neurotrophic factor (CNTF) and bFGF mainly yielded A2B5^+^/GFAP^+^ type II astrocytes, but A2B5^−^/GFAP^+^ type I astrocytes in the presence of FBS (Figures 3B,C). When exposed to thyroid hormone T3 (Rodríguez-Peña, 1999) for 5 days, all clones gave rise to MBP^+^ mature OLs with multiple interconnecting processes (Figures 3D,E; Shaw et al., 1981). No neurofilament^+^ neurons were generated in these cultures. Thus, these A2B5^+^ cells are the bona fide tripotential glial progenitor cells (GRP cells), with the potential to generate OLs and two distinct types of astrocytes (Gregori et al., 2002; Dadsetan et al., 2009; Haas et al., 2012).

Recloning experiments showed EGF and PDGFaa have a synergistic effect on the extensive self-renewal of GRP cells. After primary clones were amplified in EGF + PDGFaa for 10 days, randomly selected clones were digested into single cells and evenly plated at clonal density on three separate grid dishes, and then cultured in the presence of EGF, PDGFaa, or both. EGF and PDGFaa treatment yielded an average of 21 and 6 secondary clones, respectively. When these two factors were added together, the number of secondary clones reached an average of 51, far greater than the sum of individual factors (Figure 2D), suggesting that EGF synergized with PDGFaa in stimulating the clonal expansion of GRP cells. Secondary clones exhibited an identical pattern of antigen expressions to primary clones and can differentiate into MBP^+^ OLs, A2B5^+^ or A2B5^−^ astrocytes under corresponding environmental cues. Based on these observations, it is concluded that EGF signaling participated in the survival and extensive self-renewal of GRP cells.

### EGF Promoted the Generation of Early-Stage OPCs from GRP Clones

Tripotential GRP cells are capable of developing into bipotential OPCs under appropriate signals (Gregori et al., 2002). To investigate how EGF signaling influences this transition process, we grew freshly immunopurified GRP cells at clonal density on 48-well plates for 5 days in the presence or absence of EGF, and then analyzed the formation of OPCs from a single GRP cell by tracking clonal differentiation (Figure 4A) as described previously (Gregori et al., 2002). Since common GRP markers such as A2B5, Olig2, PDGFRα and Nestin were also positive for OPCs (Crang et al., 2004; Yang et al., 2016), only O4 monoclonal antibody can be used to define a secondary stage of OPC development (Sommer and Schachner, 1981; Chen et al., 2007; Dincman et al., 2012). In EGF group, 10.7% ± 1.3% of the clones generated O4^+^/MBP^−^ cells (Figures 4A,B). While in the control group, GRP cells were unable to divide to form clones due to the lack of growth factors, so only a few single O4^+^/MBP^−^ cells (1.3% + 0.3%) were observed (Figure 4B). The specificity of EGF signaling in promoting oligodendrogenesis was confirmed with Erlotinib HCl. When GRP cells were treated with EGF and Erlotinib HCl simultaneously, the antigenic phenotypes were similar to those of the controls (Figure 4B). Based on these results, we postulated that EGF plays a modest role in promoting the development of GRP cells into OPCs. However, it is plausible that a considerable number of clones have differentiated into O4^−^ early-stage OPCs that could not be detected immunologically. To examine this possibility, we continued to culture these single GRP cell derived clones in glial basal medium for another 5 days in the absence of EGF. In the control group, only 1.5% ± 0.2% of the cells were MBP^+^ (Figure 4D), similar to the percentage of O4^+^ clones prior to the 5-day culture (Figure 4B). In the EGF group, 32.4% ± 2.7% of the clones generated MBP^+^ cells (Figures 4C,D), suggesting that about 21.4% (32.4% MBP^+^–10.7% O4^+^MBP^−^) of the clones in EGF group generated O4^−^ OPCs. Thus, EGF predisposed GRP cells to develop along OPCs.

However, EGF treatment alone was less efficient in fully transforming GRP cells into OPCs and maintaining their O4 negative antigen phenotype. We found that GRP cells exposed to a combination of EGF + PDGFaa + T3 exhibited a significant change from fibroblast morphology to bipolar or tripolar morphology, and remained O4 negative antigen phenotype. Moreover, the vast majority of clones (89.1% ± 3.7%) generated O4^+^/MBP^+^ OLs after growth factor withdrawal. This conjecture was further strengthened by their differentiation into A2B5^+^/GFAP^+^ instead of A2B5^−^/GFAP^+^ astrocytes upon FBS treatment (Figure 4E), a bipotential differentiation characteristic of OPCs (Sommer and Schachner, 1981; Barnett et al., 1993).

### EGF Enhances the Self-Renewal of OPCs

**Figure 5:**
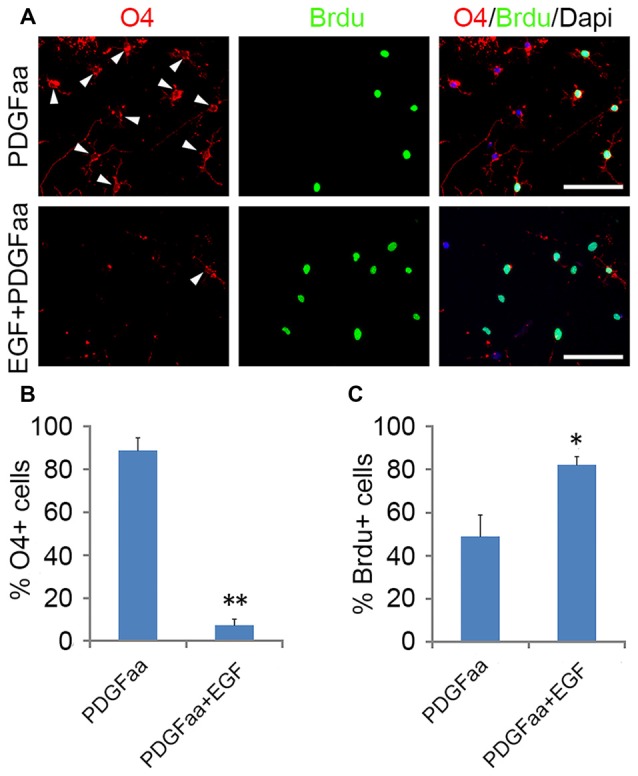
**Synergistic effect of PDGFaa and EGF in promoting the self-renewal of O4^−^ OPCs. (A)** Representative images of O4^+^ and/or BrdU^+^ cells cultured in PDGFaa or PDGFaa + EGF for 5 days, cell proliferation was analyzed by BrdU incorporation for 24 h before fixation. **(B)** Quantification of O4^+^ cells in PDGFaa and PDGFaa + EGF cultures. **(C)** Quantification of BrdU^+^ cells in PDGFaa and PDGFaa + EGF cultures. Statistical analyses are presented as mean ± SD, *n* = 3. *P < 0.05, **P < 0.01, Scale bars: 100 μm.

PDGFaa is an important mitogen for the self-renewal of OPCs (Noble et al., 1988; Raff et al., 1988; Hart et al., 1989; Neman and de Vellis, 2012), but it alone cannot maintain the O4 negative antigenic phenotype in OPCs. When GRP-derived early-stage O4^−^ OPCs from EGF + PDGFaa + T3 treatment were cultured in PDGFaa alone for 5 days, most cells became O4 positive (88.7% ± 5.9%; Figure 5A) but MBP negative with few processes. These O4^+^ late-stage OPCs (Sommer and Schachner, 1981; Gard and Pfeiffer, 1990) continued to cycle (Figures 5A,C). When EGF was present, the majority of cells maintained bipolar or tripolar morphology, and the rate of O4^+^ cells decreased substantially (7.4% ± 2.6%) with a significant increase of BrdU^+^ cells (80% ± 3.8%; Figures 5A,B). Moreover, these expanded cultures of OPCs maintained by EGF + PDGFaa can differentiate into either mature OLs or A2B5^+^/GFAP^+^ astrocytes under specific culture conditions, indicating the differentiation potential was not compromised by proliferation, nor did they revert to GRP cells. Therefore, EGF have a synergistic effect with PDGFaa in the self-renewal of O4^−^ OPCs. And this cooperative effect of EGF and PDGFaa can be further amplified by other growth factor such as bFGF. When GRP-derived early-stage O4^−^ OPCs were cultured in EGF + PDGFaa + bFGF *in vitro*, a faster cell division (87.3% ± 4.1%) and less O4^+^ cells (2.6% ± 0.9%) were observed without compromising their differentiation characteristics, suggesting that the proliferation and self-renewal of OPCs are regulated by multiple signaling pathways.

### EGF Treatment Increased the Number of Differentiated OLs

**Figure 6:**
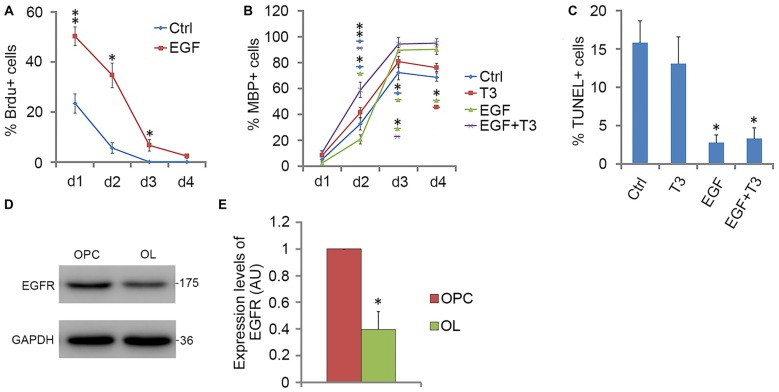
**EGF promotes OPC differentiation in cooperation with T3. (A)** Quantification of BrdU^+^ cells for OPCs cultures with EGF or not for various time lengths. **(B)** Quantification of MBP^+^ cells in OPCs differentiation cultures treated with T3, EGF or T3 + EGF for various time lengths. **(C)** Quantification of TUNEL^+^ cells in OPCs differentiation cultures on day 4. **(D,E)** Western blotting and quantitative analysis of EGFR protein expression in O4^−^ OPCs and MBP^+^ OLs, histograms express results in arbitrary units, taking GRP cells values as 100%. Statistical analyses are presented as mean ± SD, *n* = 3. *P < 0.05, **P < 0.01.

When GRP-derived O4^−^ OPCs were cultured in EGF alone, cells were initially active in proliferation, and many more BrdU-positive cells were found in EGF group than in control group. However, the percentage of BrdU^+^ cells in EGF group decreased with time, and by day 4, less than 3% of cells were proliferative (Figure 6A). Consistent with this, the EGFR expression was reduced more than half by day 4 (Figures 6D,E), which may partly contribute to the reduced effect of EGF on cell proliferation. It was found that bFGF cannot substitute for the loss of EGFR (data not shown), and the promotion of OPC proliferation by EGF or bFGF were based on the existence of PDGFaa. The reduced cell proliferation was accompanied by the increased proportion of MBP^+^ cells in culture (Figure 6B). Although the rate of OPC differentiation under EGF treatment was slightly lower than that of control and T3 groups at the beginning, it increased rapidly on day 3 and exceeded the other two groups (Figure 6B), suggesting that EGF has a significant effect in promoting OL maturation when PDGFaa is not present. A synergistic effect was observed when T3 and EGF was combined, the differentiation was faster and the efficiency was higher than that of T3 alone or EGF alone (Figure 6B). TUNEL labeling experiments revealed that the rate of apoptosis with EGF treatment was significantly lower than that of the other two groups (Figure 6C). This raised the possibility that EGF treatment may partly increase the number of mature OLs indirectly by promoting their post-mitotic survival.

## Discussion

**Figure 7:**
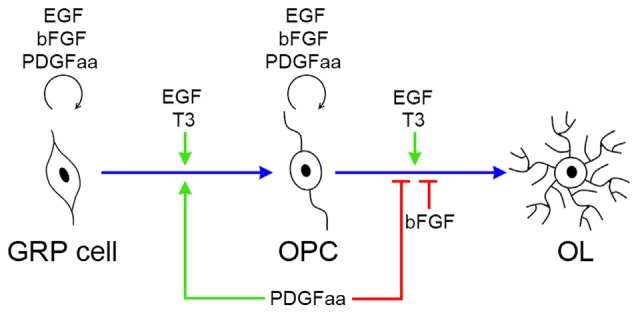
**Biological effects of EGF, PDGFaa, bFGF and T3 in the progression of OL lineage.** EGF has multiple biological effects on oligodendrogenesis, and its functional output is influenced by other signal molecules, such as PDGFaa and T3. In GRP cells, EGF and bFGF collaborate with PDGFaa to promote the self-renewal of GRP cells. When EGF is present alone, it favors the development of GRP cells to OPCs, and this progress is accelerated by supplementing PDGFaa and T3 simultaneously. In OPCs, EGF and bFGF enhance their responsiveness to PDGFaa and thus maintains their O4 negative phenotype as well as bipolar or tripolar cell morphology. When EGF is present alone, it synergizes with T3 to promote the terminal differentiation of OPCs, whereas bFGF and PDGFaa inhibit this differentiation process.

EGF is an extracellular signal molecule that binds to its specific receptor (EGFR) on the target cell membrane and then stimulates the phosphorylation of the receptor. The EGFR, also known as erbB1, is a glycoprotein that belongs to the related proteins family of c-erbB, there are three other members of this family: erbB2, erbB3 and erbB4 (Galvez-Contreras et al., 2013). The EGFR can generate homodimerization or heterodimerization with all ErbB family members, therefore, activated EGFR can stimulate a large number of downstream signaling molecules, and the complexity of its signal transduction determines the diversity of its biological effects. The diversity of EGF biological effects is reflected in the progression of OL lineage, as it plays distinct roles at different stages of OL development (Figure 7). The biological diversity of EGF signaling could be achieved by changing the balance between different signaling pathways (Tzahar et al., 1996).

### Role of EGF Signaling in GRP Development

GRP cells are probably the earliest progenitor cells for OL and astrocyte lineages (Rao et al., 1998; Gregori et al., 2002). GRP cells exhibited strong responsiveness to EGF for cell survival and proliferation in chemically defined medium. This result suggests that EGF signaling may be involved in the origin and expansion of GRP cells. Consistent with this idea, EGFR overexpression in postnatal white matter led to diffuse hyperplasia of progenitor cells that possess the same antigen phenotype as GRP cells *in vitro* (Ivkovic et al., 2008). Moreover, following lysolecithin (LPC)-induced demyelination, a significantly higher number of GRP cells was observed in EGFR-overexpressing transgenic mice than that of the wild-type, leading to faster and more extensive remyelination, and more rapid functional recovery. Conversely, reduced EGFR signaling *in vivo* decreased the generation of OLs in postnatal brains (Aguirre et al., 2007).

EGF has a variety of biological effects on GRP cells, including cell survival and proliferation, and enhanced OL lineage progression. However, its eventual functional output is influenced by other signal molecules. For instance, PDGFaa and EGF work synergistically to maintain the self-renewal, the rate of cell proliferation decreased substantially after PDGFaa withdrawal, and GRP cells progressed into OPCs. The number of secondary clones in EGF group was much lower than that of PDGFaa + EGF group, probably due to the higher differentiation rate in the EGF group, and thus the reduced ability of GRP cells to form clones.

### Role of EGF Signaling in OPC Lineage Development

OPCs retained strong responsiveness to EGF, and EGF enhanced the response of early-stage OPCs to PDGFaa in their self-renewal. In the absence of EGF, PDGFaa alone was difficult to prevent OPCs from expressing O4 antigen, and the cells then entered the secondary stage of OPC development with reduced proliferative capacity. The mechanisms underlying this enhanced response is not clear, one possibility is that it may function to maintain a high level of PDGFRα expression or signaling in OPCs (McKinnon et al., 1990). Thus, EGF may “set” a PDGF-driven clock in OPCs by establishing their sensitivity to PDGFaa (Galvez-Contreras et al., 2013). In this way, EGF may enlarge the OPC pool during CNS development.

The biological effect of EGF in promoting oligodendrogenesis was also reflected in the maturation of OPCs into OLs. EGF alone could not maintain the self-renewal of OPCs; instead it appeared to promote the differentiation of OPCs and the survival of mature OLs (Figure 6), leading to an increased number of MBP^+^ mature OLs.

### Comparison of EGF and bFGF Effects in Oligodendroglial Lineage Development

bFGF was commonly used in GRP cell culture, and it alone was sufficient to maintain the self-renewal of GRP cells *in vitro* (Gregori et al., 2002). Similarly, bFGF was also widely used in OPC culture to suppress OPC differentiation and myelin gene expression (McKinnon et al., 1990; Bansal and Pfeiffer, 1997; Decker et al., 2000; Jiang et al., 2001). Thus, it seems that bFGF promotes progenitor maintenance by preventing the lineage progression of both GRP and OPC cells.

The present study has demonstrated that EGF is also effective in the maintenance of GRP and OPC culture. However, EGF appears to enhance the self-renewal of GRPs and OPCs by enhancing their responses to PDGFRα instead of directly inhibiting their differentiation. As a matter of fact, EGF treatment alone promotes the differentiation of both GRP cells and OPCs in culture. In the absence of PDGFaa and bFGF, EGF enhances GRP lineage progression to OPCs and OPC differentiation to mature OLs in synergy with T3 (Figure 7). Therefore, EGF and bFGF possess unique and distinct roles in oligodendroglial progenitor self-renewal and differentiation. Consistent with this notion, *bFGF^−/−^* knockout leads to a higher proportion of mature OLs (Murtie et al., 2005), whereas EGFR mutation reduces the number of mature OLs (Aguirre et al., 2007).

## Author Contributions

JY designed, performed experiments, collected, analyzed the data and wrote the manuscript; XC, BX, XZ and KZ performed experiments; ZZ analyzed data; MQ designed, supervised the experiments, collected, analyzed and discussed data and wrote the manuscript. All authors listed, have made substantial, direct and intellectual contribution to the work, and approved it for publication.

## Conflict of Interest Statement

The authors declare that the research was conducted in the absence of any commercial or financial relationships that could be construed as a potential conflict of interest.

## Abbreviations

bFGF: Basic fibroblast growth factor
EGF: Epidermal growth factor
EGFR: Epidermal growth factor receptor
GRP: Glial restricted precursor
OL: Oligodendrocyte
OPC: Oligodendrocyte precursor cells
PDGFaa: Platelet derived growth factor-AA.
